# Predictors of short-term impulsive and compulsive behaviour after subthalamic stimulation in Parkinson disease

**DOI:** 10.1136/jnnp-2021-326131

**Published:** 2021-09-11

**Authors:** Anna Sauerbier, Philipp Loehrer, Stefanie T. Jost, Shania Heil, Jan N. Petry-Schmelzer, Johanna Herberg, Pia Bachon, Salima Aloui, Alexandra Gronostay, Lisa Klingelhoefer, J. Carlos Baldermann, Daniel Huys, Christopher Nimsky, Michael T. Barbe, Gereon R. Fink, Pablo Martinez-Martin, K. Ray Chaudhuri, Veerle Visser-Vandewalle, Lars Timmermann, Daniel Weintraub, Haidar S. Dafsari, EUROPAR and the International Parkinson and Movement Disorders Society Non-Motor Parkinson's Disease Study Group

**Affiliations:** 1 Institute of Psychiatry, Psychology and Neuroscience, King's College London, London, UK; 2 Department of Neurology, University of Cologne, Faculty of Medicine and University Hospital Cologne, Cologne, Germany; 3 Department of Neurology, University of Marburg and University Hospital Giessen and Marburg, Campus Marburg, Marburg, Germany; 4 Deptartment of Neurology, University of Dresden and University Hospital Dresden, Dresden, Germany; 5 Department of Psychiatry, University of Cologne, Faculty of Medicine and University Hospital Cologne, Cologne, Germany; 6 Department of Neurosurgery, University of Marburg and University Hospital Giessen and Marburg, Campus Marburg, Marburg, Germany; 7 Cognitive Neuroscience, Institute of Neuroscience and Medicine (INM-3), Research Center Jülich, Jülich, Germany; 8 Center for Networked Biomedical Research in Neurodegenerative Diseases (CIBERNED), Carlos III Institute of Health, Madrid, Spain; 9 Parkinson’s Centre of Excellence, Department of Neurology, King's College Hospital NHS Foundation Trust, London, UK; 10 NIHR Mental Health Biomedical Research Centre and Dementia Biomedical Research Unit, South London and Maudsley NHS Foundation Trust and King’s College London, London, UK; 11 Department of Stereotaxy and Functional Neurosurgery, University of Cologne and University Hospital Cologne, Cologne, Germany; 12 Departments of Psychiatry and Neurology, Perelman School of Medicine at the University of Pennsylvania, Philadelphia, Pennsylvania, USA

## Abstract

**Background:**

The effects of subthalamic stimulation (subthalamic nucleus-deep brain stimulation, STN-DBS) on impulsive and compulsive behaviours (ICB) in Parkinson’s disease (PD) are understudied.

**Objective:**

To investigate clinical predictors of STN-DBS effects on ICB.

**Methods:**

In this prospective, open-label, multicentre study in patients with PD undergoing bilateral STN-DBS, we assessed patients preoperatively and at 6-month follow-up postoperatively. Clinical scales included the Questionnaire for Impulsive-Compulsive Disorders in PD-Rating Scale (QUIP-RS), PD Questionnaire-8, Non-Motor Symptom Scale (NMSS), Unified PD Rating Scale in addition to levodopa-equivalent daily dose total (LEDD-total) and dopamine agonists (LEDD-DA). Changes at follow-up were analysed with Wilcoxon signed-rank test and corrected for multiple comparisons (Bonferroni method). We explored predictors of QUIP-RS changes using correlations and linear regressions. Finally, we dichotomised patients into ‘QUIP-RS improvement or worsening’ and analysed between-group differences.

**Results:**

We included 55 patients aged 61.7 years±8.4 with 9.8 years±4.6 PD duration. QUIP-RS cut-offs and psychiatric assessments identified patients with preoperative ICB. In patients with ICB, QUIP-RS improved significantly. However, we observed considerable interindividual variability of clinically relevant QUIP-RS outcomes as 27.3% experienced worsening and 29.1% an improvement. In post hoc analyses, higher baseline QUIP-RS and lower baseline LEDD-DA were associated with greater QUIP-RS improvements. Additionally, the ‘QUIP-RS worsening’ group had more severe baseline impairment in the NMSS attention/memory domain.

**Conclusions:**

Our results show favourable ICB outcomes in patients with higher preoperative ICB severity and lower preoperative DA doses, and worse outcomes in patients with more severe baseline attention/memory deficits. These findings emphasise the need for comprehensive non-motor and motor symptoms assessments in patients undergoing STN-DBS.

**Trial registration number:**

DRKS00006735.

## Introduction

Subthalamic nucleus (STN) deep brain stimulation (DBS) is a well-established treatment in patients with advanced Parkinson’s disease (PD), improving quality of life, motor and non-motor symptoms (NMS).^1 2^ ^3^ Specifically, STN-DBS can influence neuropsychiatric symptoms, such as depression, anxiety, alexithymia, impulsivity and compulsivity.^1 4 5^ ^6^ Currently available data on the effect of DBS on impulsive and compulsive behaviours (ICB) are contrasting, with much methodological heterogeneity.^7 8^ One of the main reported risk factors of ICB in PD is dopamine replacement therapy, particularly dopamine agonists (DA), which is possibly related to an overstimulation of the mesolimbic dopaminergic system.^9^ As STN-DBS typically leads to a significant decrease in dopaminergic medication, that is, lower levodopa equivalent daily dose (LEDD), postoperative improvement in ICB has been observed.^8 10–13^ However, studies have shown that the effect of STN-DBS on ICB is complex and goes beyond LEDD reduction, with possible adding factors including preoperative clinical aspects.^8 14^ Understanding the factors contributing to changes in ICB could be critical for improving patient selection for DBS.

Therefore, our study’s main objective was to identify clinical predictors of the effect of STN-DBS on ICB. We hypothesised that higher preoperative ICB and LEDD-DA would be predictors of beneficial postoperative ICB changes.

## Materials and methods

### Study design

This study is a prospective, observational real-life study with a 6-month follow-up including patients with PD undergoing STN-DBS. Consecutive patients were screened between August 2015 and March 2020.

### Participants

The diagnosis of PD was based on the UK Brain Bank criteria. Patients were screened for DBS treatment according to the guidelines of the International PD and Movement Disorders Society and required a sufficient levodopa responsiveness (>30% improvement in the Unified PD Rating Scale-III, UPDRS). Surgical procedures are described elsewhere.^15^

### Clinical assessment

Clinical assessments were performed at preoperative baseline (MedON) and at 6-month follow-up after DBS surgery (MedON/StimON).

Assessments included:

#### Impulsive and compulsive behaviours

The Questionnaire for Impulsive-Compulsive Disorders in PD-Rating Scale (QUIP-RS)^16^ assessed ICB during the previous 4 weeks. The QUIP-RS contains a 4×7 structure with four primary questions (about commonly reported thoughts, urges/desires and behaviours associated with ICB), each applied to seven items: (1) The first four items address impulse control disorder (ICDs) (gambling, buying, sexual and eating behaviours), (2) Items 5 and 6 assess other compulsive behaviours (punding and hobbyism) and (3) Item 7 assesses compulsive medication use. In each of the four primary questions, all seven items are scored on a five-point Likert scale from 0 (never) to 4 (very often). Therefore, the QUIP-RS total score ranges from 0 to 112. To ascertain if the reported symptoms were clinically relevant, we used the previously published cut-off scores.^17^ The cut-off for ICDs (items 1–4) was a total score ≥10, and for other compulsive behaviours (items 5 and 6) ≥7. A cut-off score for compulsive medication use has not been established yet, due to its low prevalence in some PD populations.^17^

Additionally, expert psychiatrists specialised in examinations of patients with PD interviewed patients focusing on ICB.

Secondary outcomes included:

#### Quality of life

PD Questionnaire-8 (PDQ-8) is a well-established tool to measure quality of life in patients with PD and is commonly used in patients undergoing DBS.^18^ ^19 20^ Furthermore, it is recommended by the International Parkinson and Movement Disorder Society.^21^ The data are expressed as PDQ-8 Summary Index (SI) ranging from 0 (no impairment) to 100 (worst level).^22 23^

#### Non-motor symptoms

The clinician-rated NMS Scale (NMSS) contains 30 items divided into nine domains: (1) cardiovascular, (2) sleep/fatigue, (3) mood/apathy, (4) perceptual problems/hallucinations, (5) attention/memory, (6) gastrointestinal tract, (7) urinary, (8) sexual function and (9) miscellaneous (including pain, inability to smell/taste, weight changes, and sweating). Symptoms over the last 4 weeks are assessed. The NMSS total score ranges from 0 (no NMS) to 360 (maximum NMS impairment).^24 25^

#### Motor symptoms

- The Hoehn and Yahr (HY) scale classifies the severity of motor symptoms into five stages, reflecting disease progression and deterioration and ranges from 0 (no signs of disease) to 5 (needing a wheelchair or bedridden unless assisted).^26^
- The UPDRS domains II, III and IV assess activities of daily living, motor evaluation and motor complications. The UPDRS domains range from 0 (no impairment) to 52, 108 and 23 respectively.^27^

### Statistical analysis

Gaussian distribution of test scores was assessed using the Shapiro-Wilk method. Wilcoxon signed-rank tests, respectively, paired t-tests, if parametric test criteria were fulfilled, were employed to test for changes at 6-month follow-up. This analysis was performed in the overall cohort and the subgroup of patients who experienced clinically relevant baseline ICB. We corrected for multiple comparisons using the Bonferroni method and report adjusted p-values at the significance threshold of 0.05. Post hoc, the relationship between preoperative clinical outcome parameters and QUIP-RS score changes were explored using Spearman correlations. The QUIP-RS change score (mean Test_baseline_ – mean Test_follow-up_) was correlated with the following baseline variables: age, disease duration since diagnosis, QUIP-RS, PDQ-8 SI, NMSS total score, NMSS domains, UPDRS part II to IV, LEDD total and LEDD-DA. The correlations were graded between 0.0 and 0.19 ‘very weak’, 0.20–0.39 ‘weak’ 0.40–0.59 ‘moderate’, 0.60–0.79 ‘strong’ and 0.80–1.0 ‘very strong’.^1^ Positive correlations indicate that higher baseline values are associated with more postoperative QUIP-RS improvement.

To identify clinical predictors of ICB after STN-DBS, we analysed stepwise linear regressions. We included QUIP-RS change score as the criterion variable and parameters from the correlation analyses with a relaxed threshold (p<0.25) as candidate predictor variables.^28^ Multicollinearity was checked using intercorrelations between candidate predictor variables (r>0.6) and variance inflation factors, which should not exceed 10.^29^

To confirm the feasibility of identified predictors and explore between-group differences, we dichotomised the QUIP-RS changes and defined groups of patients experiencing a clinically relevant ‘QUIP-RS improvement’ and ‘QUIP-RS worsening’ based on a designated threshold of ½ SD of QUIP-RS total at baseline. This cohort-derived threshold was used in several previous studies.^1 30^ Patients who experienced no clinically relevant improvement or worsening of QUIP-RS changes were not included in this analysis. Differences between these two groups were tested using Mann-Whitney U tests.

All analyses were conducted using SPSS V.26.0 for Mac.

## Results

In total, 55 patients (69.1% male) were included with a mean age of 61.7 years (±8.5 SD) and a mean disease duration of 9.8 years (±4.6 SD). The median baseline HY stage was 2 (IQR 2–3).

### Pre-DBS and post-DBS clinical characteristics

At baseline, 38.9% of patients reported ICB. Among those, 16.2% reported eating disorders, 5.4% hypersexuality, 5.4% excessive shopping, none gambling problems and 31.4% reported punding and hobbyism.

Clinical characteristics at baseline and 6-month follow-up are presented in table 1.

**Table 1:** Clinical characteristics at baseline and follow-up

Overall cohort	N	Baseline	FU	P value
		Mean (SD)	Mean (SD)	
QUIP-RS total	55	15.4 (13.1)	14.6 (13.5)	0.585
PDQ-8 SI	51	29.3 (17.4)	21.2 (14.3)	**0.005**
NMSS total	55	53.8 (29.9)	35.5 (25.4)	**<0.001**
UPDRS				
Part-II	54	11.7 (6.7)	7.7 (5.4)	**<0.001**
Part-III	50	21.3 (10.5)	18.3 (11.0)	0.199
Part-IV	54	6.7 (3.7)	3.6 (3.2)	**<0.001**
LEDD total	55	1102.4 (444.5)	578.1 (317.3)	**<0.001**
LEDD-DA	54	248.1 (162.7)	164.8 (127.1)	**<0.001**
Patients with clinically relevant preoperative impulsive and compulsive behaviour				
QUIP-RS total	14	30.5 (10.7)	24.1 (14.0)	**0.044**
PDQ-8 SI	14	36.2 (12.4)	24.8 (11.0)	0.054
NMSS total	14	74.7 (37.9)	43.3 (26.2)	**0.016**
UPDRS				
Part-II	13	12.5 (8.7)	7.7 (5.6)	n.s.
Part-III	13	20.6 (14.7)	15.8 (7.8)	n.s.
Part-IV	13	8.7 (3.2)	3.8 (3.8)	0.058
LEDD total	14	1178.1 (519.6)	576.4 (316.2)	**0.004**
LEDD-DA	14	280.8 (101.8)	157.5 (101.0)	**0.010**

Wilcoxon signed-rank or t-tests, when parametric test criteria were fulfilled, between baseline and 6-month follow-up to analyse within-group changes of outcome parameters.

Bold font highlights significant results.

Multiple comparisons (two groups) were corrected with the Bonferroni method.

FU, follow-up; LEDD, levodopa equivalent daily dose; LEDD-DA, LEDD of dopamine agonists; NMSS, Non-Motor Symptom Scale; n.s., not significant; PDQ-8 SI, Parkinson’s Disease Questionnaire-8 Summary Index; QUIP-RS, Questionnaire for Impulsive-Compulsive Disorders in Parkinson’s Disease Rating Scale; UPDRS, Unified Parkinson’s Disease Rating Scale.

In the overall cohort, the main outcome QUIP-RS total score did not change at follow-up. However, in the subgroup of patients reporting clinically relevant preoperative ICB, the QUIP-RS total improved significantly at follow-up (30.5±10.7 before vs 24.1±14.0 afterward, p=0.044).

In the overall cohort, secondary outcomes including PDQ-8 SI, NMSS total, UPDRS-II, UPDRS-IV, HY, LEDD total and LEDD-DA improved significantly from baseline to 6-month follow-up. In the subgroup of patients experiencing preoperative ICB, secondary outcomes including NMSS total, LEDD total and LEDD-DA improved significantly and a trend was observed for PDQ-8 SI and UPDRS-IV. In contrast to the overall cohort, the UPDRS-II outcome was not significant.

### Correlation analyses

In the overall cohort, Spearman correlations between baseline clinical outcome parameters and the QUIP-RS change score resulted in significant correlations for the higher baseline QUIP-RS total score (r_s_=0.454, p=0.001; ‘moderate’) and the lower baseline LEDD-DA (r_s_=−0.351, p=0.009; ‘weak’). There were no significant correlations between the QUIP-RS change score and baseline NMSS mood/apathy domain (all p>0.05). QUIP-RS change score was not significantly correlated with LEDD total or LEDD-DA changes in the overall cohort and in patients with ICB at baseline (all p>0.05). Exploring the relationship of LEDD-DA changes and preoperative NMSS domain scores, we found no significant correlations with the attention/memory and mood/apathy domain scores (all p>0.05).

### Predictor analysis

Univariate linear regression analyses with change in QUIP-RS total score as the criterion variable were performed using candidate predictor variables identified in correlation analyses (relaxed threshold p<0.25).^28^ Besides LEDD-DA and QUIP-RS total score, at the relaxed inclusion threshold, this included the NMSS attention/memory domain (r_s_=−0.187, p=0.171) and UPDRS-III (r_s_=−0.212, p=0.139).

Significant predictor variables were the QUIP-RS total score (β=0.353, p=0.008) and LEDD-DA (β=−0.342, p=0.010). The model accounted for 22.4% of the variance ( $R^{2}=0.224,$ in QUIP-RS change $\left(F_{2,46}=7.936;p§amp;lt;0.001\right)$ .

### Difference between ‘QUIP-RS improvement’ and ‘QUIP-RS worsening’ groups

The cut-off for a clinically relevant change in QUIP-RS total score was 6.55 points according to the designated threshold ½ SD of baseline QUIP-RS total score. Out of 55 patients in the overall cohort, 16 patients (29.1%) experienced a clinically relevant improvement and 15 patients (27.3%) a clinically relevant worsening in the QUIP-RS total score. In 24 patients (43.6%), ICB symptoms were stable.

In the overall cohort, in the ‘QUIP-RS worsening’ group compared with the ‘QUIP-RS improvement’ group, we observed at baseline higher LEDD-DA (321.9 mg ±139.2 vs 180.3 mg ±156.1, p=0.021), a lower QUIP-RS total (12.1±13.6 vs 21.8±10.6, p=0.009) and higher NMSS attention/memory domain scores (5.1±4.4 vs 2.8±5.1, p=0.043).

In the cohort of 14 patients reporting clinically relevant preoperative ICB, a clinically relevant improvement was observed in six patients (median baseline QUIP-RS score: 29.5, IQR: 21.5–40.25) and worsening in one patient (median baseline QUIP-RS score: 48), whereas QUIP-RS changes remained stable in seven patients (median baseline QUIP-RS score 24.0, IQR: 20–36).

## Discussion

In the present study, we assessed clinical aspects predicting ICB in 55 patients with PD undergoing STN-DBS. The QUIP-RS total score improved significantly in patients experiencing preoperative ICB. Furthermore, we observed considerable inter-individual variability of QUIP-RS outcomes as 27.3% of patients experienced a clinically relevant worsening and 29.1% a clinically relevant improvement. Post hoc analyses revealed that higher baseline QUIP-RS and lower baseline LEDD-DA were associated with greater QUIP-RS improvements.

### Quality of life, NMS and motor symptoms, and medication requirements in the overall cohort

Following previous studies, we observed a postoperative improvement of quality of life, non-motor, and motor symptoms, and reduced total dopaminergic medication and DA in the overall cohort.^1 31 32^

### Impulsive and compulsive behaviour

#### Prevalence and severity

In our cohort, 39% of patients reported ICB at baseline, with hobbyism and punding being the most frequent, followed by eating disorders, excessive shopping and hypersexuality. These findings agree with previous studies in DBS populations.^5 33 34^ Moreover, as in previous studies, we observed considerable interindividual variability of postoperative changes of ICB. There was no linear trend with 27.3% of patients experiencing a clinically relevant worsening and 29.1% a clinically relevant improvement.^12 14 33^

To our knowledge, this is among one of the first studies analysing a wide range of motor and non-motor predictors for ICB. We found that a higher QUIP-RS total score is the strongest predictor for greater improvements in postoperative QUIP-RS score. Notably, we found a significant improvement in the QUIP-RS in patients reporting clinically relevant preoperative ICB. A study by Rossi *et al* reported a trend for QUIP-RS improvement in patients with higher preoperative ICB. The significance threshold may have been missed in that study as they included a smaller cohort size (n=37).^33^ In contrast, Moum *et al* reported that preoperative ICB resolved postoperatively in 2/7 patients, whereas 17 patients developed newly diagnosed ICB postoperatively.^35^ However, this was only a retrospective analysis, and the study cohort was not homogeneous as unilaterally and bilaterally implanted patients in different DBS targets (STN and globus pallidus internus) were included.

#### Motor symptoms and dopaminergic medication

Consistent with previous studies, we did not find an effect of the preoperative motor score (UPDRS-III) on postoperative ICB changes.^8^

Moreover, we found that lower LEDD-DA was a predictor for greater improvement in QUIP-RS total score. A possible explanation might be that patients with higher tendency to develop ICB, may be unable to tolerate higher doses of DA,^36 37^ perhaps prompting surgical evaluation. However, the change in LEDD total and LEDD-DA was not associated with a change in QUIP-RS total score, even in the population with ICB at baseline. This lack of association may result from a non-linear relationship of LEDD and severity of ICB, which may be based on patient-specific thresholds of the dopaminergic medication causing ICB. Previous studies reported the missing correlation between LEDD changes and ICB outcome, which underlines that the DBS effect on ICB appears not solely to be based on dopaminergic treatment.

#### Attention/memory and other NMS

Furthermore, the present study is the first to report a relationship between more severe preoperative attention/memory deficits and a clinically relevant postoperative worsening of ICB. This finding suggests that neuropsychological deficits are detrimental to ICB outcomes. This result complements a recent multicentre study reporting more severe ICB in demented compared with non-demented patients with PD.^38^ Our study expands the observations by Kim *et al* who reported in a cross-sectional analysis more severe cognitive impairment (worse Mini-Mental State Examination score) in patients with worsened or de novo development of ICB retrospectively assessed with the QUIP-RS after STN-DBS. However, in contrast to this study, we found no significant association between higher preoperative mood disorders assessed with the NMSS mood/apathy domain and greater improvement of impulsivity and compulsivity. Kim *et al* used the Beck Depression Inventory, which might be more sensitive to smaller differences in depression symptoms than the NMSS, which we used in the present study. Furthermore, a recent study by des Neiges Satin *et al* reported a higher risk of developing ICD post STN-DBS in patients with apathy preoperatively, a finding that we did not confirm with our data.^39^ The authors used the Ardouin Scale of Behaviour which might be more sensitive to capture apathy than the NMSS.

Our group’s previous study provided evidence for a ‘sweet spot’ for beneficial effects of STN-DBS on attention/memory,^4^ and Mosley *et al* report the connectivity profile of DBS effects on impulsivity and compulsivity.^5^ Assuming the underlying pathomechanisms of these symptoms are connected through common neural correlates, future studies are needed to investigate common connectivity profiles of STN-DBS effects on attention, memory, mood, impulsivity and compulsivity. This information may help DBS programming in patients with concomitant preoperative neuropsychological symptoms and ICB.

### Limitations

There are several limitations to this study. ICBs, which are not caused by dopaminergic medication, are considered a contraindication for DBS, and our results cannot be applied to patients experiencing these. Furthermore, our study’s population size was relatively small with 55 patients, few of which experienced clinically relevant ICB at baseline or clinically relevant postoperative improvement or worsening of ICB. Follow-up assessments in the present study were conducted 6 months after surgery. Previous studies have shown that ICB can occur at long-term follow-up in patients treated with DA.^40^ Further studies are warranted to address the long-term effects of STN-DBS on ICB. The multicentre design of our study is likely to reduce the potential bias of a single-centre design. In this study, we did not analyse DBS settings or volume of tissue activated by DBS, as we were interested in preoperative clinical predictors of ICD outcomes. Furthermore, caregiver reports were not assessed no additional behavioural tasks were investigated to corroborate verbal QUIP-RS reports. The QUIP-RS surveys symptoms over the previous 4 weeks, including motor ON and OFF states. In this analysis, we opted to calculate a cut-off value for the classification of clinically relevant ‘QUIP-RS improvement’ and ‘QUIP-RS worsening’ based on a previously published cohort-specific method (½ SD of test_baseline_). To our knowledge, minimal clinically significant differences have not yet been determined for the QUIP-RS. We used a distribution-based method because DBS cohorts include highly selected patient populations, resulting in considerable differences of baseline ICB compared with the general population of patients with PD.^12^ Distribution-based methods seek to determine minimal clinically important differences based on the observed scores and, therefore, they simply express the change in a standardised way.^41^ A disadvantage of the anchor-based methods is the possibility that the minimal clinically important difference falls within the instrument’s random variation and the susceptibility of some ratings to recall bias.^42 43^ Furthermore, future studies are needed to investigate if neuroimaging of the dopaminergic and possibly also the cholinergic system may provide objective biomarkers for the identification of patients who are at risk of developing ICB, in particular when concomitant attention/memory deficits are observed.

## Conclusion

In this study, we observed favourable ICB outcomes in patients with higher preoperative ICB burden and lower doses of DA, whereas more severe preoperative attention/memory deficits were associated with clinically relevant ICB worsening after STN-DBS. This study’s novel findings highlight the importance of a comprehensive assessment of patients’ motor and non-motor profiles before DBS surgery. Further studies in larger cohorts analysing a wide range of motor and NMS may better predict patients’ postoperative risk of developing ICDs. The overall aim of this line of research is a better selection of patients for DBS therapy.^44^

## Data Availability

Data are available on reasonable request.
